# Successfully treated synchronous double malignancy of the breast and esophagus: a case report

**DOI:** 10.1186/1752-1947-4-169

**Published:** 2010-06-03

**Authors:** Abhishek Singh, Ishwar Chandra Khare, Awadhesh Kumar Dixit, Kailash Chandra Pandey, Deepak Kumar Mittal, Parul Singh

**Affiliations:** 1 Department of Radiation and Clinical Oncology, Swami Rama Cancer Institute, UFHT Medical College, Haldwani, India; 2 Department of Radiotherapy, JK Cancer Institute, GSVM Medical College, Kanpur, India; 3 Department of Ophthalmology, Swami Rama Cancer Institute, UFHT Medical College, Haldwani, India

## Abstract

**Introduction:**

The incidence of multiple primary cancers is reported to be between 0.3% and 4.3%. The second primary lesion is identified either simultaneously with the primary lesion (synchronous) or after a period of time (metachronous). Few cases of metastasis of breast carcinoma to the esophagus and vice versa have been reported in the past.

**Case presentation:**

We report an extremely rare case of a 55-year-old Indian woman who had carcinomas in both the esophagus and the breast simultaneously. She was treated successfully using combined modalities of surgery, chemotherapy and radiation therapy.

**Conclusion:**

Cases of synchronous double malignancies can be treated by dealing with the malignancy in the two sites as independent carcinomas. We have to take into consideration the total dose of radiation to a critical organ as well as the effect of the total dose of toxic chemotherapeutic drugs on our patient.

## Introduction

The incidence of double malignancy is very low, as is a case of synchronous breast and esophageal carcinomas. Double malignancy cases pose the problem of finding the best treatment for the patient. We present such a case which was treated successfully.

## Case presentation

A 55-year-old Indian woman reported to the hospital with complaints of dysphagia for solid foods for more than one year which had progressively increased in severity. At presentation, she had also developed difficulty in swallowing liquids and had a history of regurgitation of food after meals. There was no history of cough or difficulty in breathing during meals, thus ruling out the possibility of a tracheoesophageal fistula.

A thorough physical examination revealed a lump in the left breast of approximately 3 × 3 cm in size. The lump was hard in consistency with irregular margins, and it was not fixed to the skin or to underlying structures. Two firm, mobile ipsilateral axillary lymph nodes with mild tenderness could be palpated. Our patient did not have any family history of breast or ovarian carcinoma. She had breastfed all three of her children and had been postmenopausal for eight years. There was also no history of oral contraceptive pills or hormone replacement therapy.

Upper gastrointestinal endoscopy revealed a friable, ulceronodular lesion at the gastroesophageal junction involving the juxta-esophageal fundus. Endoscopic biopsy of the lesion was carried out. Histopathological examination of the biopsy tissue showed moderately differentiated squamous cell carcinoma. A computed tomography (CT) scan of our patient's thorax and abdomen showed a soft tissue density space-occupying lesion in the distal esophagus and cardiac end of her stomach. The lesion was characterized as an irregular thickening of the wall with a widened lumen. The length of involvement was approximately 6 cm.

There was no proximal dilation of the esophagus. Evidence of aspiration was seen in the right basal segment of her lung. No atelectasis or pneumanitic lung tissue was seen. Significantly sized bronchopulmonary lymphadenopathy was seen bilaterally.. Anterior and superior mediastinal facial planes were preserved. No significant anteroposterior or superior-mediastinal lymph nodes were found. No pleural collection of fluid was seen. An increased attenuating lesion was seen in the left breast superior to the nipple, measuring approximately 24 mm in diameter. The margins of this lesion were not sharply defined, and no calcification was seen. No other lesions were found. Her liver was mildly enlarged with no focal lesions and a normal portal venous system and intra-hepatic biliary radicles. The porta hepatis was also free of lymph nodes.

Significant lymphadenopathy was seen along the celiac trunk and the lesser curvature of the stomach along the gastric artery. The para-aortic and para-caval regions were normal. The splenic hilum was also free of lymph nodes. All other findings were within normal limits.

Fine needle aspiration cytology from the lump of the left breast suggested an infiltrating ductal carcinoma. In view of our findings, a transhiatal esophagogastrectomy and a left-sided modified radical mastectomy with axillary dissection were carried out. Ten centimeters of the esophagus as well as 5 cm along the lesser and 3 cm along the greater curvature of the proximal stomach were removed. Grossly, the tumor involved the adventitia of the lower end of the esophagus and also the pericardial fat.

**Figure 1:**
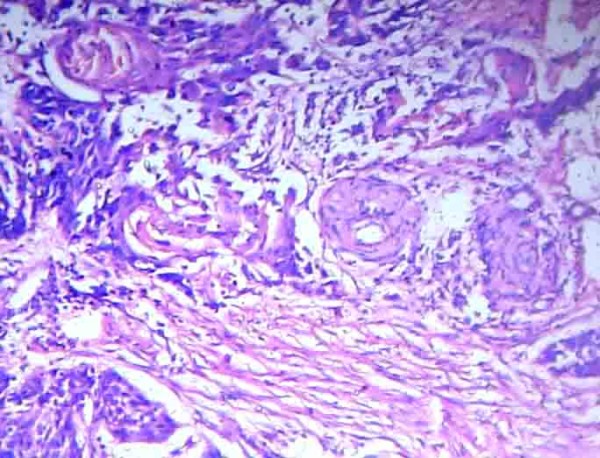
**Histopathology of esophageal growth**. Photomicrograph of histopathological analysis of esophageal growth showing moderately differentiated squamous cell carcinoma type with a predominantly pseudo-glandular pattern.

Microscopic analysis showed moderately differentiated squamous cell carcinoma type with a predominantly pseudo glandular pattern, involving the lower third of the esophagus, the cardioesophageal junction and the cardiac end of the stomach (Figure 1). The tumor had invaded the muscular layer of the esophagus and extended to the adventitia. Perineural infiltration and lymphatic emboli were noted. Circumferential cut margins were free of tumor. The tumor had infiltrated the full thickness of the wall of the cardia of the stomach and had invaded the peri-gastric fat. Seven out of nine nodes along the lesser curvature showed metastasis with perinodal extension. One node along the greater curvature showed metastasis with perinodal extension. Esophageal cut margin and gastric cut margin were free of tumor.

**Figure 2:**
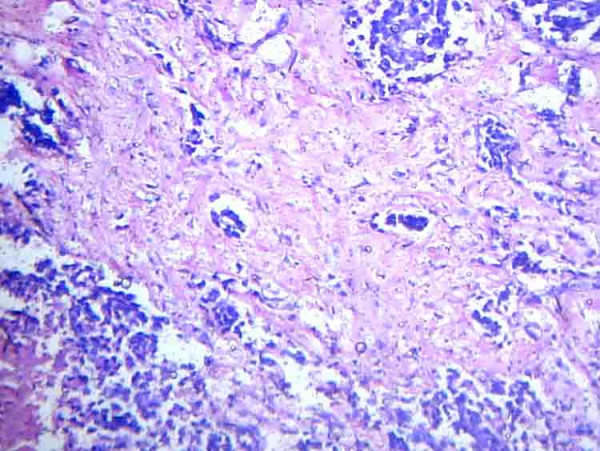
**Histopathology of breast lesion**. Photomicrograph of histopathological analysis of left modified radical mastectomy specimen showing infiltrating ductal carcinoma grade II.

Histopathological analysis of the left modified radical mastectomy revealed the tumor to be an infiltrating ductal carcinoma grade II (Figure 2). A comedo-type of ductal carcinoma *in situ *of nuclear grade II was noted within the same tumor, the content of which was not clinically significant. Areas of necrosis and tumor calcification were noted. No perineural infiltration and lymphovascular emboli were found.

The nipple, areola, skin and base were free of tumor. All the 14 lymph nodes we dissected were reactive. Immunohistochemistry of the operative specimens was performed. The breast tumor was negative for estrogen and progesterone receptors, but was positive for human epidermal growth factor receptor 2 (HER-2 neu).

**Figure 3:**
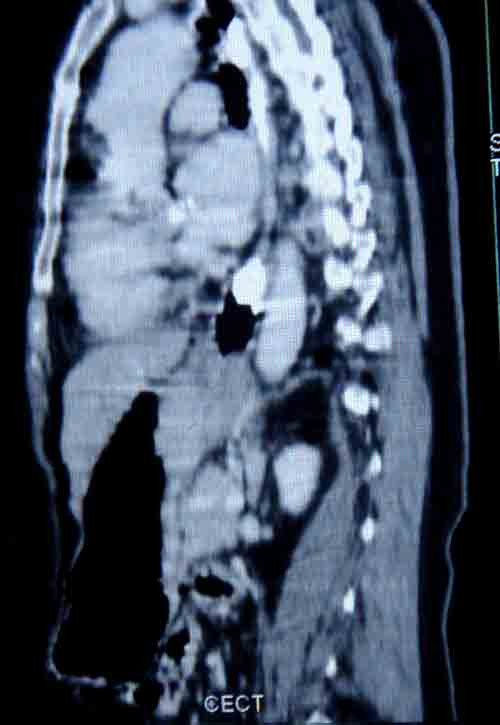
**Computed tomography of thorax showing recurrence of growth in the lower part of the esophagus**.

Three months after surgery, our patient complained of dysphagia. A CT scan revealed a recurrence of the carcinoma in her esophagus (Figure 3). Our patient had received four cycles of docetaxel and Adriamycin (doxorubicin)-based chemotherapy before the recurrence of the esophageal growth. As our patient was HER-2neu positive, a taxane-based regimen was planned, considering that the single agent docetaxel has previously been effective in treating esophageal carcinomas. Our patient was not prescribed Herceptin (trastuzumab) as she could not afford the drug. Instead, she was treated with locoregional radiation therapy for the recurrence of the carcinoma in her esophagus, taking care with the dosage delivered to the heart. She was given a total dose of 65Gy in 32 fractions over seven weeks. Initially, 40Gy was given by anterioposterior-posterioanterior fields. The remaining dose was delivered by three oblique fields. The remaining two cycles of chemotherapy were given after completion of the radiation treatment. Our patient is currently symptom-free and doing well one year and eight months after completion of the treatment. She had follow-up clinical examination monthly for one year. An upper gastrointestinal endoscopy and a CT scan of her thorax were performed every three months. Currently, our patient has follow-up appointments every two months and is advised to have six-monthly investigations.

## Discussion

The incidence of multiple primary cancers is rare and is reported to be between 0.3% and 4.3%. The second primary lesion is identified either simultaneously with the primary lesion (synchronous) or after a period of time (metachronous). The association between synchronous primary tumors in the aerodigestive tract is a well-known phenomenon that has been explained by the concept of "field cancerization" [1]. The mucous epithelium of the head and neck, lung and esophagus is exposed to common carcinogenic agents, leading to multiple carcinomas in these regions. Strong epidemiological evidence implicates tobacco as the main carcinogen and alcohol as a promoter of carcinogenesis. The incidence of synchronous cancers in patients with esophageal cancer ranges from 3.6% to 27.1% [2,3]. Most of these synchronous cancers are in the head and neck regions.

Other frequently reported sites of synchronous cancer associated with esophageal cancer are the stomach, lung and urinary bladder [4,5]. Although numerous epidemiological studies over more than five decades have pointed to drinking and smoking as possible causes of this phenomenon [2-4], the reason why some patients are particularly likely to develop multiple cancers remains obscure. Cases of malignancy of the breast with synchronous or metachronous malignancies of the ovary, stomach, rectum and oral cavity have been reported. Studies have shown the association of both adenocarcinoma and squamous cell carcinoma of the esophagus with carcinoma of the breast [6]. There have also been some reports of carcinoma of the esophagus in women who were treated successfully for carcinoma of the breast, but none of the cases reported had both malignancies simultaneously. Besides these, there have been few case reports of metastasis of breast carcinoma to the esophagus or vice versa.

No other cases of synchronous double malignancy of breast and esophageal carcinomas can be found. Breast carcinoma represents one of the most common origins of metastasis to the esophagus [7]. The common sites of metastasis from breast carcinoma include local and distant lymph nodes, lung parenchyma, bones, liver and brain. Though less common, gastrointestinal carcinomas involving everything from the tip of the tongue to the rectum, secondary to metastatic breast carcinoma, have been reported. Most of these lesions occur years after treatment of the primary breast cancer and can be confused with a second primary. There have also been reports of esophageal carcinoma developing after radiation treatment to the primary breast carcinoma. Here, we report an extremely rare case of a patient having carcinoma of the esophagus and the breast at the same time. In this case, the histopathology of the breast tumor was an infiltrating ductal carcinoma grade II and the histopathology of the esophageal growth was a moderately differentiated squamous cell carcinoma type, ruling out any possibility of metastasis from any one site to the other.

Resection of both neoplasms frequently offers the best chance of long-term survival. However, even in the case of an incurable synchronous cancer (e.g. metastatic prostate cancer), esophagectomy is not always contraindicated [8]. The type of treatment for such esophageal carcinomas strongly depends on the type and prognosis of the synchronous malignancy. Evidence-based arguments about whether to perform a simultaneous or a staged operation are not available. Studies report that simultaneous resection of both neoplasms has acceptably low morbidity and mortality rates [9]. However, for each patient, the risks and benefits of simultaneous surgery should be weighed against those of a staged surgery [10].

Our patient underwent surgery for both the primaries because her general condition was good and she had no other medical reasons to deny surgery. However, the choice of chemotherapy was a difficult task. She was given four cycles of Adriamycin (doxorubicin) and docetaxel-based chemotherapy given that HER-2/neu positive breast carcinomas respond better to taxane-based chemotherapy and docetaxel is effective as a single agent treatment for esophageal carcinoma. Radiation therapy was given to treat a recurrence in our patient's esophagus. Two more cycles were administered after the completion of radiation treatment. Studies of multiple primary malignancies have been useful tools for exploring risk factors by examining associations between different malignancies. An association between two cancers might suggest that those cancers share etiological risk factors. Three tumor suppressor genes common to breast and esophageal carcinomas are p53, Rb and p16 genes. Two risk factors that are common to these two carcinomas are alcohol intake and obesity.

Positive association between alcohol intake and carcinoma of the breast has been consistently demonstrated. The risk appears to be linearly related to the amount of alcohol consumed. Alcohol intake and tobacco use are considered to be the major contributory factors in the development of esophageal carcinoma worldwide. It is estimated that 90% of squamous cell carcinoma of the esophagus in western Europe and North America can be attributed to tobacco and alcohol use.

About 25% of breast carcinomas worldwide are due to obesity, according to the international agency for research on cancer. The literature indicates that increased body mass index (BMI) is a risk factor for adenocarcinoma of the esophagus and that individuals with the highest BMI have up to a seven-fold greater risk of esophageal carcinoma than those with a low BMI. Other possible explanations for such an association would be a hereditary predisposition to multiple cancers, as side effect of previous treatment for cancer, or to a chance phenomenon. Further studies are needed to guide the treatment of such cases.

## Conclusions

The incidence of multiple primary cancers is rare. The reason why some patients are more prone to develop multiple cancers remains obscure. Synchronous double malignancies can be treated by considering the malignancy at two separate sites as independent carcinomas, taking in consideration the total dose of radiation to a critical organ and the total dose of toxic chemotherapeutic drugs.

## Consent

Written informed consent was obtained from the patient for publication of this case report and the accompanying images. A copy of the written consent is available for review by the Editor-in-Chief of this journal.

## Competing interests

The authors declare that they have no competing interests.

## Authors' contributions

AS was involved in the conception, design, analysis and interpretation of data, and the draft for the version to be published. ICK was involved in the conception, design, interpretation of data, and provided inputs for important intellectual contents. AKD and KCP contributed in drafting the manuscript or revising it critically for important intellectual content. DKM and PS were involved in the acquisition of data, analysis and interpretation of data, and provided inputs for important intellectual content. All authors read and approved the final manuscript.
